# Excessive Weight Gain during the First Year of Peritoneal Dialysis Is Associated with Inflammation, Diabetes Mellitus, and a Rapid Decrease in Residual Renal Function

**DOI:** 10.1371/journal.pone.0139033

**Published:** 2015-09-25

**Authors:** Jwa-Kyung Kim, Young-Su Kim, Young Rim Song, Hyung Jik Kim, Sung Gyun Kim, Sung Jin Moon

**Affiliations:** 1 Department of Internal Medicine, Kidney Research Institute, Hallym University Sacred Heart Hospital, Hallym University, Anyang, Korea; 2 Department of Internal Medicine, International St. Mary’s Hospital, Catholic Kwandong University College of Medicine, Incheon, Korea; Hospital Universitario de La Princesa, SPAIN

## Abstract

**Objectives:**

Significant weight gain is a potential problem in most patients starting peritoneal dialysis (PD); however, few studies have explored the clinical effects of increased body weight (BW) in these patients. We evaluated the effect of excess weight gain during the first year after PD on residual renal function (RRF).

**Methods:**

A total of 148 incident PD patients were analyzed in a longitudinal observational study. The mean duration of follow-up was 23.8 months. RRF was measured at baseline (within 1 month of starting PD) and thereafter at 6-month intervals for 2–3 years or until loss of RRF. BW was measured at the time of RRF measurement, and excess weight gain was defined as a BW increase over the median value (3.0%).

**Results:**

The median 1-year increase in BW was 2.3kg (IQR, 1.01–4.58) or 3.0% (IQR, 1.13–5.31). The mean slope of RRF decline was –0.068 ± 0.053 mL/min/month/1.73m^2^, and RRF loss developed in 48 patients at a mean follow-up time of 19.4 ± 6.8 months. Patients with BW increases > 3.0% showed significantly increased RRF decline rate compared to those without excess weight gain (p<0.001), and the BW increase (%/year) correlated significantly with higher hs-CRP levels and RRF decline rate. High systolic blood pressure, diabetes, large amount of proteinuria and excess BW gain significantly influenced the RRF decline rate. Also, it increased the risk of RRF loss by 4.17-fold (95% confidence intervals, 1.87–9.28; p<0.001).

**Conclusions:**

Excess weight gain during the first year of PD was closely linked to systemic inflammation, diabetes and rapid decline in RRF.

## Introduction

Patients with end-stage renal disease (ESRD) generally experience significant weight gain after initiating peritoneal dialysis (PD), and a large proportion of PD patients become obese during PD treatments [1]. Diaz-Buxo et al. reported that significant body weight (BW) gain occurred during the first 17 months after initiating PD; the mean BW gain was 6.4% [2], attributable largely due to an increased caloric intake secondary to dialysate glucose absorption. A previous bioimpedance analysis (BIA) study evaluating 1-year changes in body composition found that incident PD patients experienced significant increases in total body fat and visceral fat mass. The changes were more marked than in incident hemodialysis (HD) patients. [3–6]. Visceral adiposity is regarded as an independent predictor of cardiovascular (CV) disease by worsening arterial stiffness and endothelial dysfunction in this population [7]. An increase in body fat mass may be a major driver of systemic inflammation and adverse long-term outcomes in PD patients [8].

In the general population, obesity and increased visceral fat mass are closely linked to higher morbidity and mortality [9]; however, any adverse role played by obesity in ESRD patients remains unclear. Although a high body mass index (BMI) is associated with improved survival in HD patients (the “reverse epidemiology” of ESRD [10,11]), conflicting results have been obtained in PD patients [12–15].

We thus assessed the effect of excess weight gain within the first year after PD on residual renal function (RRF), one of the most important prognostic factors of survival in PD patients.

## Methods

### Study population and data collection

This observational cohort study consisted of 177 patients who initiated PD between January 2007 and January 2013. We excluded patients who received HD more than two weeks before the start of PD (n = 3), those transferred to another PD center (n = 6), those who switched to HD or received a kidney transplant within 12 months after PD (n = 10), patients with daily urine output < 200 mL at the start of PD (n = 4), and those who underwent RRF measurements less than twice (n = 6). A total of 148 patients we ultimately analyzed.

The data collected included age, sex, underlying cause of renal disease, comorbidities, duration of PD therapy, systolic blood pressure (SBP), and concurrent drug prescriptions. Baseline BMI was calculated as BW/(height/100)^2^, and obesity was defined as a baseline BMI ≥ 25 kg/m^2^. The levels of serum albumin, cholesterol, creatinine, and normalized protein nitrogen appearance (nPNA), were measured to estimate nutritional status. Baseline proteinuria was measured using spot urine protein-to-creatinine ratio (UPCR), and each RRF was calculated as the mean of the sum of 24-h urea and creatinine clearance. To estimate the rate of RRF decline, RRF was measured at baseline (within 1 month of starting PD) and thereafter at 6-month intervals for 2–3 years or until loss of RRF. Loss of RRF was defined as urine volume < 100 mL/day. The median number of RRF measurements was five (interquartile range, [IQR], 4–6). We also calculated average daily glucose absorption. This depends on dextrose concentration, the peritoneal membrane transport rate, length of dwell, and the number of exchanges, and thus varies widely. Usually, each exchange was associated with absorption of 60–70% glucose in CAPD patients and 30–50% in APD patients. Therefore, in the present study, we calculated daily total glucose absorption (for each exchange) as follows: sum of glucose concentrations (13.6, 22.5 and 38.6 g/L for 1.5%, 2.5% and 4.25%, respectively) X dwell volume (L) X 0.7 (CAPD) or 0.5 (APD). BW measurements were performed at the time of RRF measurement. Excess weight gain was defined as an increase in BW that was greater than the median value (3.0%) of all patients. The changes in BW during the first year of PD was calculated as follows: change in BW (%) = [(BW at 1 year after PD—BW at baseline)/BW at baseline] × 100. Peritonitis was considered present when at least two of the following three findings were made: abdominal pain, a cloudy effluent, and/or an effluent WBC count over 100/μL with at least 50% neutrophils. Peritonitis rates are reported as numbers of episodes per patient-year (PY) of follow-up.

### Outcomes

The primary outcome of this study was to compare the slope of RRF decline by excess weight gain during the first year of PD. The secondary outcome was to evaluate the effect of excess weight gain on RRF loss.

### Ethical issues

This study was approved by the local Institutional Review Board/Ethics Committee of Hallym University Sacred Heart Hospital, and all study procedures adhered to the Declaration of Helsinki. As the work was a retrospective observational cohort study using data obtained during routine practice in our PD center, we did not obtained informed consent. However, we asked all patients, in advance, if their medical records could be used for research purposes and all agreed.

### Statistical analysis

Statistical analyses were performed using SPSS ver. 21.0 (SPSS Inc., Chicago, IL, USA). All variables were expressed as means ± standard deviations or medians with ranges. The Kolmogorov—Smirnov test was used to analyze normality of the data distribution. Natural log values were used for skewed data in multivariate analysis, such as baseline RRF, proteinuria, and high sensitivity-C reactive protein (hs-CRP). The slope of RRF decline over time was determined by linear regression analysis of serial urinary urea and creatinine measurements for each patient and was expressed as the regression coefficient (mL/min/month/1.73m^2^). Patients were divided into two subgroups according to the median value of BW gain (3.0%) during the first year of PD, and differences between these two groups were analyzed by independent *t*-test or Mann—Whitney *U*-test for continuous variables or the χ^2^ test for categorical data. Longitudinal changes in RRF due to excess BW gain were evaluated using repeated measures analysis of variance (ANOVA), and the missing data was analyzed by the last observation carried forward method. Significant factors associated with the rate of RRF decline and loss of RRF were analyzed by multivariate linear regression and the Cox proportional hazard model, respectively. A p value <0.05 was considered to indicate statistical significance.

## Results

### Baseline characteristics of the patients

The baseline characteristics of the 148 patients are shown in Table 1. Mean age was 54.2 ± 11.9 years. Ninety (60.8%) patients were male, and mean follow-up duration was 23.8 ± 9.7 months. Mean BMI was 23.8 ± 3.2 kg/m^2^, and the prevalence of obesity was 38.5%. The median RRF at the start of PD was 5.02 (2.59–6.00) mL/min/1.73m^2^ and the overall rate of RRF decline was –0.068 ± 0.053 mL/min/month/1.73m^2^. The median increase in weight during the first year of PD was 2.3 kg (IQR, 1.01–4.58) or 3.0% (IQR, 1.13–5.31).

**Table 1:** Baseline clinical and biochemical characteristics by excess BW gain. Abbreviations: BMI; body mass index, WBC; white blood cell, HDL: high-density lipoprotein, LDL; low-density lipoprotein; hs-CRP; high-sensitivity C-reactive protein.

		BW increase during first year		
Clinical characteristics	Total	>3.0%	≤3.0%	P
	(n = 148)	(n = 75, 50.7%)	(n = 73, 49.3%)	
RRF decline rate	-0.068 ± 0.053	-0.092 ± 0.049	-0.048 ± 0.045	<0.001
Age (years)	54.2 ± 11.9	54.5 ± 10.4	53.8 ± 12.9	0.739
Gender, male (%)	90 (60.8)	47 (62.7)	43 (58.9)	0.396
BMI (kg/m^2^)	23.8 ± 3.2	23.5 ± 3.2	24.0 ± 3.3	0.385
BMI ≥ 25 kg/m^2^, n (%)	57 (38.5)	31 (41.3)	26 (35.6)	0.293
Systolic blood pressure (mmHg)	142.5 ± 21.4	142.6 ± 22.3	142.4 ± 20.6	0.748
Diabetes, n (%)	81 (54.7)	48 (64.0)	33 (45.2)	0.016
Baseline laboratory findings				
WBC (/uL)	6896 ± 2030	7240 ± 2278	6566 ± 1697	0.036
hemoglobin (g/dL)	9.0 ± 1.4	9.2 ± 1.4	8.9 ± 1.3	0.101
serum albumin (g/dL)	3.4 ± 0.6	3.3 ± 0.6	3.4 ± 0.5	0.134
serum glucose (mg/dL)	141.2 ± 64.3	149.8 ± 71.9	130.7 ± 54.5	0.060
HDL- cholesterol (mg/dL)	43.1 ± 13.7	42.3 ± 11.4	43.8 ± 14.4	0.496
LDL- cholesterol (mg/dL)	103.7 ± 44.9	106.4 ± 46.7	101.5 ± 42.5	0.533
triglyceride (mg/dL)	134.8 ± 72.0	136.9 ± 77.6	132.5 ± 53.0	0.718
ln_hs-CRP	0.08 ± 1.06	0.27 ± 0.89	-0.18 ± 1.17	0.012
ln_urine PCR	0.58 ± 0.93	0.85 ± 0.84	0.31 ± 0.95	<0.001
Medication, n (%)				
ARB/ACEi	123 (83.1)	61 (81.8)	62 (84.9)	0.358
Calcium channel blocker	86 (58.1)	42 (56.0)	44 (60.3)	0.616
Statin	67 (45.3)	38 (50.7)	29 (39.7)	0.121
Diuretics	98 (66.2)	53 (70.7)	45 (61.6)	0.162
RRF at the start of dialysis	5.02 (2.59–6.00)	4.76 (2.57–6.00)	5.28 (3.16–7.00)	0.171
(mL/min/1.73m^2^)*†				

After 1 year of PD therapy, the mean BMI had increased to 24.6 ± 3.5 kg/m^2^, and the prevalence of obesity to 46.6% (n = 69).

The prevalence of diabetes, white blood cell count (WBC), ln hs-CRP, ln UPCR levels were higher in patients with BW gain >3.0%. However, age, BMI, SBP, other laboratory findings such as hemoglobin, glucose and cholesterol, and medication use were similar between patients with or without excess BW gain. Of PD-related parameters, average daily glucose absorption was significantly higher in patients with excess weight gain. Other parameters including the daily urine volume, the D/P creatinine 4 hour, peritoneal equilibration test (PET) data, and peritonitis rate were comparable between the two groups (Table 2).

**Table 2:** Comparison of baseline PD-related parameters by excess BW increase. RRF; residual renal function, D/P creatinine; dialysate/plasma creatinine ratio, nPNA; normalized protein nitrogen appearance, PET; peritoneal equilibration test, PY; patient-year.

		BW increase during first year		
Clinical characteristics	Total	>3.0%	Clinical	Total
	(n = 148)	(n = 75, 50.7%)	characteristics	(n = 148)
urine volume (mL/day)	1005 ± 610	963.5 ± 573.6	1050.8 ± 644.9	0.391
D/P creatinine, 4 hour	0.72 ± 0.16	0.71 ± 0.13	0.71 ± 0.18	0.466
Kt/V				
- total	2.28 ± 0.72	2.26 ± 0.85	2.30 ± 0.56	0.717
- peritoneal	1.47 ± 0.61	1.52 ± 0.72	1.42 ± 0.48	0.339
- renal	0.83 ± 0.55	0.76 ± 0.56	0.88 ± 0.54	0.364
Ultrafiltration (mL)	476.2 ± 105.6	506.6 ± 99.2	444.1 ± 133.9	0.281
Glucose absorption (g/day)	104.5.± 36.1	111.4 ± 41.3	97.8 ± 31.0	0.022
nPNA (g/kg/day)	0.97 ± 0.52	1.02 ± 0.71	0.93 ± 0.22	0.238
PET				0.164
- high or high-average	68 (45.9)	31 (41.3)	37 (50.7)	
- low or low-average	80 (54.1)	44 (58.7)	36 (49.3)	
Peritonitis rate (number/PY)	0.55 ± 0.93	0.62 ± 0.72	0.47 ± 1.10	0.327

**Fig 1:**
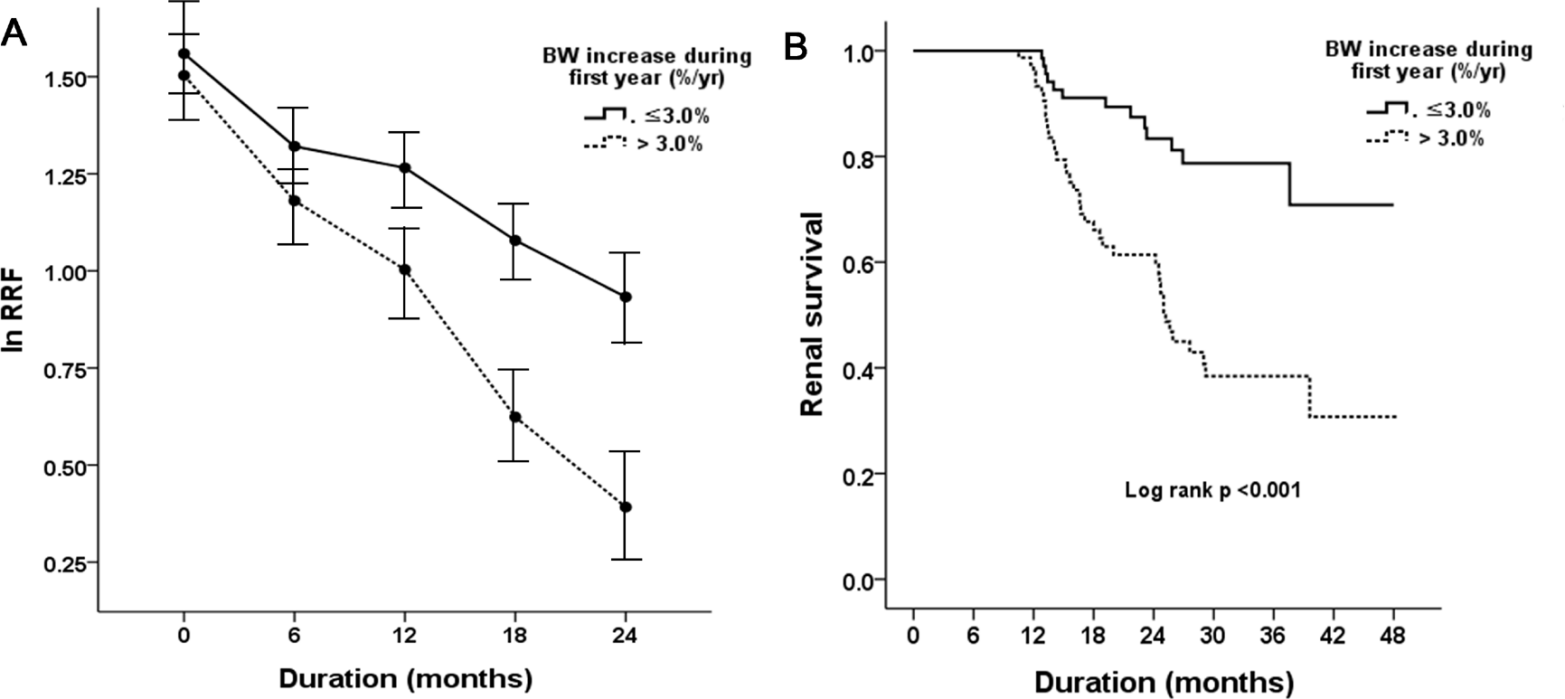
(A) Effect of excess BW gain on the log RRF value. RRF decline was significantly accelerated in patients exhibiting an excess BW gain (a BW increase > 3.0% during the first year of PD). (B) Kaplan-Meier estimates of RRF loss. Excess BW gain was a significant risk factor for such loss.

The RRF decline rate of patients who gained excess BW was more marked than that of patients who did not gain excess BW (-0.092 ± 0.049 vs -0.048 ± 0.045 mL/min/month/1.73m^2^, p <0.001). Fig 1A shows the effect of excess BW gain on the rate of RRF decline. Patients with BW gain >3.0% had significantly higher rates of RRF decline. A significant interaction was observed between RRF decline rate and 1-year BW gain (within-subjects effects: F = 6.124, p<0.001; between-subjects effect: F = 4.487, *p* = 0.037) when analyzed by repeated-measures ANOVA.

### Correlation analysis of the RRF decline rate and BW increase

The RRF decline rate correlated with the ln UPCR (r = −0.460, p < 0.001), followed by BW gain during the first year of PD (r = −0.407, p < 0.001), diabetes (r = −0.362, p < 0.001), systolic BP (r = −0.276, p = 0.001), ln hs-CRP (r = −0.228, p = 0.009), HDL-cholesterol (r = 0.174, p = 0.037), RRF at the start of PD (r = 0.145, p = 0.043) and peritonitis rate (r = −0.172, p = 0.036). In addition, the amount of BW increase during the first year was significantly associated with the extent of glucose absorption, WBC count, ln UPCR, and ln hs-CRP.

### Factors associated with RRF decline and loss of RRF

Multivariate linear regression showed that increases in BW and systolic BP, diabetes, and ln UPCR were significant determinants of the rate of RRF decline (Table 3). In subgroup analysis by diabetes status, a BW increase had a significant association with rapid RRF decline rate, irrespective of diabetes (diabetes group, β = -0.182, p = 0.046 and non-diabetes, β = -0.380, p = 0.001, respectively).

**Table 3:** Factors associated with RRF decline: Multivariate linear regression analysis.

RRF decline rate (mL/min/1.73m^2^/mo)		Adjusted*		
	β	B	95% CI for B	P value
**Total patients**				
SBP, mmHg	-0.160	-0.001	-0.002, -0.001	0.029
Diabetes	-0.178	-0.019	-0.035, -0.003	0.020
ln_UPCR (g/g)	-0.247	-0.013	-0.021, -0.005	0.001
BW increase (%)	-0.300	-0.004	-0.006, -0.002	0.001
ln_hsCRP	-0.118	-0.007	-0.012, 0.001	0.103
**Diabetes only**				
SBP, mmHg	-0.277	-0.001	-0.002, -0.001	0.008
ln_UPCR (g/g)	-0.351	-0.022	-0.035, -0.009	0.001
BW increase (%)	-0.182	-0.002	-0.006, -0.001	0.046
ln_hsCRP	-0.152	-0.009	-0.018, 0.001	0.072
**Non-diabetes only**				
SBP, mmHg	-0.095	-0.001	-0.001, 0.000	0.342
ln_UPCR (g/g)	-0.206	-0.009	-0.018, -0.001	0.048
BW increase (%)	-0.380	-0.007	-0.011, -0.005	0.001
ln_hsCRP	-0.177	-0.009	-0.016, 0.002	0.152

*adjusted for age, gender, SBP, BMI, diabetes, serum HDL level, UPCR, hs-CRP levels, RRF at baseline, BW increase (%), glucose absorption, and peritonitis rate.

During follow-up, RRF loss was observed in 48 participants (32.4%) over a mean duration of 19.4 ± 6.8 months. Kaplan—Meier analysis showed significant differences in RRF loss by BW gain (Log rank p<0.001, Fig 1B). In an unadjusted Cox’s proportional hazard model, all of SBP, diabetes, RRF at baseline, ln UPCR, the peritonitis rate, and excess BW gain, were significant risk factors for loss of RRF. Even after adjustments of significant variables, excess BW gain increased the risk of RRF loss by 4.17-fold (95% confidence interval, 1.87–9.28; p<0.001) (Table 4). Similarly in subgroup analysis, excess BW gain was a significant risk factor for loss of RRF, irrespective of diabetes, (S1 Table).

**Table 4:** Factors predicting RRF loss in incident PD patients. Abbreviations: SBP; systolic blood pressure; BMI; body mass index, RRF; residual renal function, UPCR; urine protein-to-creatinine ratio.

	Unadjusted		Adjusted*	
Factors	Hazard ratio (95% CI)	P	Hazard ratio (95% CI)	P
Age (per 1year)	1.01 (0.98–1.03)	0.643	-	-
Gender (male/female)	1.49 (0.81–2.75)	0.198	-	-
SBP (per 10 mmHg)	1.20 (1.09–1.41)	0.004	1.01 (0.99–1.02)	0.174
Diabetes (presence)	3.13 (1.62–6.03)	0.001	2.06 (1.03–4.11)	0.040
BMI (per 1 kg/m^2^)	0.96 (0.88–1.05)	0.383	-	-
RRF at baseline (per 1 mL/min/1.73m^2^)	0.77 (0.64–0.87)	<0.001	0.80 (0.69–0.94)	0.006
ln UPCR (per 1 g/g)	1.93 (1.41–2.63)	<0.001	1.69 (1.16–2.5)	0.006
Excess BW gain (presence)	6.04 (2.82–12.93)	<0.001	4.17 (1.87–9.28)	<0.001
Peritonitis rate (number/yr)	2.43 (1.36–4.36)	0.003	1.28 (0.97–1.72)	0.077

* adjusted for SBP, diabetes, UPCR, RRF at baseline, peritonitis rate and an excess BW gain.

## Discussion

In this longitudinal observational study, we evaluated the effect of excess BW gain during the first year of PD on the rate of RRF decline. We found that BW increased by a mean of 3% during the first year of PD. Also, excess weight gain (>3.0%) was closely associated with a rapid decline in RRF, and increased the risk for RRF loss by 4.17-fold. This is the first study to evaluate the effect of excess BW gain during the first year of PD on RRF.

In both incident and prevalent PD patients, significant weight gain is inevitable because of an increased caloric intake secondary to dialysate glucose absorption and improvement of protein energy wasting caused by uremia. Although a previous multicenter study with 1,911 incident Brazilian PD patients found that BW gain during the first year of PD therapy did not increase mortality, the study duration was relatively short, and changes in RRF were not considered.[16]

The precise mechanisms underlying the close relationship between BW gain and rapid RRF decline remain unclear. However, we suggest that chronic systemic inflammation may be in play. In the present study, patients exhibiting excess BW gain had significantly higher WBC counts and hs-CRP levels at baseline. High baseline hs-CRP levels were strongly associated with significant BW increase and rapid RRF decline. Moreover, weight gain associated with fat accumulation may aggravate the oxidative stress and inflammation, implicated in the pathogenesis of progressive atherosclerosis. Indeed, obesity is considered to be a state of chronic inflammation [17], and visceral adiposity is an independent predictor of cardiovascular (CV) disease by worsening arterial stiffness and endothelial dysfunction in dialysis population [7,18].

Furthermore, in the present study, large amount of proteinuria also correlated with significant BW gain and a rapid decline in RRF. Previous experimental and clinical evidence suggests that proteinuria accelerates the progression of kidney disease through multiple pathways, including induction of tubular chemokine expression and complement activation, which lead to inflammatory cell infiltration into the interstitium and sustained fibrogenesis [19,20]. Moreover, proteinuria could be a sign of systemic inflammation that leads to a poor prognosis and CV mortality. Trimarchi et al. showed that nephrotic range proteinuria is associated with systemic inflammation and cardiac stress independent of fluid overload or removal in chronic HD patients [21]. However, the relationship between a large amount of proteinuria and increased BW or fat accumulation in PD patients may be rather complex. Although it is well established that increased BMI or obesity is a risk factor for the development of proteinuria [22,23], any inverse association, namely, the effect of proteinuria on obesity or fat accumulation has not yet been demonstrated.

Another interesting finding is that BW increase was closely associated with the glucose absorption level at baseline, and patients with excess BW gain showed significantly higher glucose absorption than those without. Although higher-level glucose absorption may increase BW, reverse association is also possible. As the excess BW group exhibited higher-level baseline proteinuria, dialysates of higher glucose concentrations may be needed to control edema at the start of PD. Therefore, this finding should be interpreted with caution. A causal relationship between the extent of glucose absorption and BW increase has not yet been identified.

On the other hand, any increase in BW after initiation of PD could be caused by a decline in RRF. In general, as the duration of dialysis is extended, RRF decreases further, triggering edema that could be associated with BW gain. However, in the present study, the median time to loss of RRF was 19.0 months. Therefore, we think that excess BW gain during the first year after PD treatments may be not the result of, but the cause of RRF decline.

We suggest that the BW increase observed in the present study may be attributable to a rise in fat mass. However, as we did not perform BIA, we could not directly demonstrate changes in body composition during the first year of PD. This is a limitation of our study. However, we tried to clinically control edema in PD patients, and previous studies found that BW gain after PD is likely due to an accumulation of body fat [4–6]. Although some proportion of BW increase may be attributable to a rise in body water level, volume overload per se may exert harmful effects on the RRF on PD patients. Inal S et al. reported that the extracellular water to total body water ratio, a marker of volume overload, was a risk factor for left ventricular mass index (LVMI). Additionally, the mean LVMI negatively correlated with daily urine volume [24]. Therefore, regardless of whether the BW increase is caused by fat accumulation or fluid overload, such an increase may accelerate RRF decline.

Finally, the prognostic effect of diabetes on RRF decline rate was very clear in our study. In addition to BW gain, the presence of diabetes significantly predicted rapid RRF decline and RRF loss. In this context, the role of excess BW increase was less evident in diabetic subgroup, suggesting that diabetes-associated large amount of proteinuria and inflammation may be important in determining RRF decline. Supporting our findings, with nationally representative sample of US adults (n = 74,710), Jackson et al found that the BMI—mortality relationships differed markedly by diabetes status. In adults with diabetes, BMI was significantly, but inversely, associated with mortality. In non-diabetic patients, however, BMI was positively associated with mortality; a “J curve” was apparent. The cited authors considered that, in diabetic patients, an “obesity paradox” might be in play, associated with protein energy wasting and associated frailty and distress [25].

Our study has several limitations. First, this was a retrospective, single-center work, with a relatively small sample size. Further large-scale multicenter studies are needed to reproduce our data. Second, we did not perform body composition analyses, so we could not confirm that BW gain was caused by an increase in fat mass. In future, studies measuring fat mass and volume status using BIA and serological markers, like NT-pro BNP, are warranted. Third, we sought to identify associations between the decrease in RRF and inflammation or oxidative stress. However, we did not measure specific inflammatory cytokines such as interleukin-6 and tumor necrosis factor-α, or markers of oxidative stress.

In conclusion, most patients starting PD experienced significant weight gain during the first year. Excessive weight gain during the first year of peritoneal dialysis is associated with inflammation, diabetes, and rapid decrease of RRF. Therefore, more attention should be paid to changes in BW to halt the decrease in RRF in incident PD patients.

## Supporting Information

S1 Table The role of excess BW gain in predicting RRF loss by the presence of diabetes. (DOC)
