# Supplementary material for: Excessive Weight Gain during the First Year of Peritoneal Dialysis Is Associated with Inflammation, Diabetes Mellitus, and a Rapid Decrease in Residual Renal Function
Source: PLoS One. 2015 Sep 25;10(9):e0139033. doi: 10.1371/journal.pone.0139033 (PMC4583287; doi:10.1371/journal.pone.0139033)
Supplement: S1 Table — (DOC) [file pone.0139033.s001.doc]

**Supplementary Table 1.** The role of excess BW gain in predicting RRF loss by the presence of diabetes.

| Factors | Diabetes | | | |  | Non-diabetes | | | |
| --- | --- | --- | --- | --- | --- | --- | --- | --- | --- |
|  | Univariate | | Multivariate | |  | Univariate | | Multivariate | |
|  | Hazard ratio (95% CI) | P | Hazard ratio (95% CI) | P |  | Hazard ratio (95% CI) | P | Hazard ratio (95% CI) | P |
| Age (per 1year) | 0.99(0.96-1.03) | 0.561 | - | - |  | 1.02 (0.97-1.06) | 0.504 | - | - |
| Gender (male/female) | 1.13 (0.57-2.24) | 0.717 | - | - |  | 3.73 (0.82-2.95) | 0.089 | - | - |
| SBP (per 10 mmHg) | 1.02 (1.01-1.04) | 0.004 | 1.01 (0.99-1.03) | 0.239 |  | 1.04 (0.98-1.03) | 0.784 | - | - |
| BMI (per 1 kg/m^2^) | 0.87 (0.78-1.15) | 0.473 | - | - |  | 1.06 (0.72-1.38) | 0.422 | - | - |
| RRF at baseline | 0.78 (0.64-0.95) | 0.015 | 0.81 (0.67-0.97) | 0.023 |  | 0.72 (0.51-0.99) | 0.040 | 0.71 (0.52-0.96) | 0.030 |
| ln UPCR (per 1 g/g) | 1.97 (1.33-2.93) | <0.001 | 2.04 (1.24-3.35) | 0.005 |  | 1.69 (1.11-3.03) | 0.017 | 0.93 (0.50-1.63) | 0.833 |
| Excess BW gain (presence) | 1.20 (1.10-1.32) | 0.001 | 2.87 (1.18-6.96) | 0.019 |  | 1.28 (1.13-1.46) | <0.001 | 7.56 (2.00-15.73) | 0.010 |
| Peritonitis rate (number/yr) | 1.14 (0.93-1.31) | 0.073 | 1.27 (0.92-1.81) | 0.147 |  | 3.43 (1.81-6.36) | 0.001 | 2.16 (0.68-6.85) | 0.227 |
